# Differences in demographics and clinical outcomes in older, middle-aged, and younger adults with low back pain receiving chiropractic care

**DOI:** 10.1186/s12998-025-00589-w

**Published:** 2025-07-31

**Authors:** L. A. Hansen, J. Hartvigsen, R. K. Jensen

**Affiliations:** 1 Chiropractic Knowledge Hub, Campusvej 55, 5230 Odense M, Denmark; 2 Center for Muscle and Joint Health, Department of Sports Science and Clinical Biomechanics, University of Southern Denmark, Campusvej 55, 5230 Odense M, Denmark

**Keywords:** Low back pain, Older adults, Chiropractic care, Disability, Musculoskeletal comorbidities, Depression, Age differences, Background

## Abstract

**Background:**

The evidence on age-related differences in outcomes in patients seeking chiropractic care for low back pain is limited. The aims of this study were to (i) to explore differences in patient characteristics and symptoms between older, middle-aged and younger patients with LBP seeking chiropractic care, (ii) to investigate whether age was associated with changes in physical function at 2, 13 and 52 weeks follow-up and (iii) to evaluate if other specific demographic variables were associated with changes in physical function over time.

**Methods:**

This observational cohort study (November 2016 to December 2018) used data from the Danish Chiropractic Low Back Pain Cohort (ChiCo). Participants ≥ 18 years seeking chiropractic care for new onset low back pain were categorised into three age groups: young adults (< 40 years), middle-aged adults (40–59 years), and older adults (≥ 60 years). Disability was assessed at baseline and at 2, 13 and 52 weeks follow-up using the Roland Morris Disability Questionnaire. Associations between age groups and disability outcomes were analysed using linear regression, while associations with demographics, and social and psychological factors were examined using backward stepwise linear regression.

**Results:**

2777 participants were included. At baseline, there were no significant differences in disability scores between age groups. Older patients reported more non-musculoskeletal comorbidities and prescription pain medication use, compared to younger and middle-aged patients. Younger patients showed higher depression and anxiety levels compared to middle-aged and older patients. Younger and middle-aged patients more frequently reported multiple musculoskeletal comorbidities than older patients. At all follow-ups, older patients had slightly higher disability scores and showed less improvement over time compared to younger and middle-aged patients, indicating a modest association between age and poorer outcomes. Higher baseline disability, more musculoskeletal comorbidities, worse self-reported health, and higher depression scores were more consistently associated with less improvement in disability over time.

**Conclusion:**

Older chiropractic patients with low back pain had slightly higher disability scores compared to younger and middle-aged patients, but age was not the strongest factor associated with disability outcomes. Baseline disability, depression, self-perceived general health, and MSK comorbidities were more consistently linked to higher disability scores across all follow-up time points.

**Supplementary Information:**

The online version contains supplementary material available at 10.1186/s12998-025-00589-w.

## Background

The proportion of people aged 60 years and over are expected to increase from 12 to 22% globally between 2015 and 2050 according to the World Health Organization [1]. This shift will lead to more age-related conditions [1], including cardiovascular disease, cognitive decline and musculoskeletal (MSK) conditions such as low back pain (LBP) [2]. LBP is currently the leading cause of disability in most countries and affects all age groups, but is more prevalent in older adults [3]. The expected rise in life expectancy suggests an increase in the overall burden of disability and associated economic costs associated with LBP [3].

Older adults with LBP experience more severe pain that has greater impact on daily activities and physical function compared with younger patients [4–7]. At the same time, they have higher prevalence of comorbidities, including other MSK conditions[7, 8] which negatively affect physical activity and physical function [9]. Older adults with LBP are also at high risk of psychological distress, such as depression, anxiety and kinesiophobia, which correlate with less favourable outcomes in pain and physical function [10–16]. Finally, older adults are more likely to report longer recovery times than younger adults [17].

Clinical guidelines recommend non-pharmacological interventions for the management of non-specific LBP in older adults, focusing on physical therapies, psychological support and lifestyle modification [2]. However, older adults with LBP are more likely to be prescribed analgesics only and less likely to receive physiotherapy, exercise recommendations, or specialist referral compared with younger patients [6]. This suggests that older patients may not be receiving guideline compliant management.

Chiropractors, who provide guideline-recommended non-pharmacological interventions to improve physical function, may help to address this gap in evidence-based care for older adults with LBP. Previous research found that chiropractic care for older people seems to be both safe and beneficial [18]. However, older adults are less likely to seek chiropractic care compared to younger adults. In Denmark in 2023 for example only 20% of patients in chiropractic practice were aged 65 or older [19]. Similarly, the COAST and O-COAST studies found that just 12% of chiropractic patients in Australia (COAST) [20] and 19% in Canada (O-COAST) [21] were aged 65 or older. This underrepresentation highlights the need to better understand the characteristics and treatment outcomes of older patients who do seek chiropractic care.

The aims of this study were (i) to explore differences in patient characteristics and symptoms between older, middle-aged and younger patients with LBP seeking chiropractic care, (ii) to investigate whether age was associated with changes in physical function at 2, 13 and 52 weeks follow-up and (iii) to evaluate how other demographic variables were associated with changes in physical function over time.

## Methods

### Design

This was an observational cohort study based on data from the Danish Chiropractic low back pain Cohort (ChiCo) [22]. The study was reported according to the general recommendations for observational studies, Strengthening the Reporting of Observational studies in Epidemiology (STROBE) [23].

### Setting

Patients seeking chiropractic care for a new episode of LBP with or without leg pain between November 2016 and December 2018 were recruited from 10 medium or large sized private chiropractic clinics in the Central Denmark Region. The data from questionnaires were collected and stored in the electronic research database REDCap, managed by the Odense Patient data Explorative Network, OPEN [22].

Participating chiropractic clinics received a small reimbursement (DKK 225 per included patient) to compensate for the additional workload related to recruitment and data collection [22]

### Participants

Participants had to be 18 years or older, have new onset LBP with or without leg pain, and be able to complete electronic questionnaires in Danish. A ‘new onset of LBP’ was defined as a recent or recurrent episode of LBP for which the patient was not receiving treatment or long-term management. Patients referred for immediate surgical evaluation or suspected of having underlying conditions requiring further diagnostic evaluation were not included in the study. No incentives or financial compensation were provided to study participants. Details of the cohort have been described elsewhere [22].

### Data

Patients that were found eligible at the initial visit were given an iPad on arrival, on which they were informed about the project and gave their consent to participate. After consent, they completed the Baseline 1 questionnaire before the chiropractic consultation, followed by the Baseline 2 questionnaire after the consultation. Follow-up questionnaires were sent by email at 2, 13 and 52 weeks. Non-responders received email reminders, followed by telephone contact from a research assistant if necessary. Details of the data collection as well as data access, storage and protection, have been described previously [22]

### Variables

The following variables were collected from the Baseline 1 questionnaire:

### Age and sex

Patient age in years were calculated from the Central Person Register (CPR) number, a unique personal identification number assigned to all people residing in Denmark, and categorized into ‘Young adults’ (< 40 years), ‘Middle-aged adults’ (40–59 years), and ‘Older adults’ (≥ 60 years).

Patient sex (male or female) was also gleaned from the CPR number.

### Disability

Disability was answered in the Baseline 1 questionnaire and at 2, 13 and 52 weeks follow up. It was measured using the 23-item Danish version of the Roland Morris Disability Questionnaire (RMDQ), converted to a score of 0–100 (higher scores indicate higher levels of disability) [24–26].

### Pain

Back- and leg pain was measured on a numerical rating scale (NRS) from 0–10 [27, 28], and answered in the Baseline 1 questionnaire.

### Psychological factors

Depression and anxiety were evaluated using two questions from the Danish version of the Örebro Musculoskeletal Pain Screening Questionnaire (OMPQ) [29], based on the PICKUP model [30]. Depression was assessed by: *“*How much have you been bothered by feeling depressed in the past week?” and anxiety by: “How tense or anxious have you felt in the past week?”, both rated on a 10-point scale (0 = Not at all/Absolutely calm, 10 = Extremely/Very tense) [31].

Fear of movement/kinesiophobia was measured using two items from the Tampa Scale for Kinesiophobia (TSK) [32]. “I’m afraid that I might injure myself if I exercise” and “Pain always means I have injured my body.” Responses on a 4-point scale (Strongly disagree to Strongly agree) were summed and dichotomized into High and Low risk of kinesiophobia [32].

The following variables were collected from the Baseline 2 questionnaire.

### Social factors

Educational level was assessed by the question “What is your longest education?” with eight possible answers, categorised into the following four groups: 1 ‘No education/Primary school/Youth education’, 2 ‘Vocational education/Short further education’, 3 ‘Middle further education’, and 4 ‘Higher education’. The last category ‘Other’ was omitted due to limited responses.

Cohabitation was assessed by the question “Do you live with a spouse/partner?” (No/Yes).

Self-perceived general health was measured on a five-point scale from ‘excellent’ to ‘poor’.

### Body mass index (BMI)

BMI was calculated from the patient’s height and weight and then categorised as ‘Normal weight (BMI 18,5–24,9)’, ‘Overweight (BMI 25–29,9)’ or ‘Obese (BMI > 30)’. ‘Underweight’ (BMI < 18.5) was not included due to the limited number of patients in this category.

### Comorbidities

Non-MSK comorbidities were assessed in the baseline questionnaire with the question: “Did a doctor ever tell you that you have or have had…?”, followed by a list of 15 diseases (Supplementary file 1, Table S1). MSK-comorbidities were assessed with: “In addition to LBP, have you experienced…?” in the past two weeks, listing 13 potential pain sites (Supplementary file 1, Table S2). Comorbidities were categorized as 0, 1, 2, or 3 + MSK or non-MSK comorbidities.

### Analgesics

Use of analgesics for back pain were evaluated by the question: Do you currently take medication for your low back pain? With answers No, Yes—prescription medication or yes—non-prescription medication.

### Statistical methods

Descriptive data for the whole population and for each age group (< 40, 40–59, and ≥ 60) were presented as proportions for categorical variables and as means and confidence intervals, or median and interquartile interval (IQI) for continuous variables depending on the distribution of data. Group comparisons were conducted using Pearson’s chi-square test for categorical variables and either the t-test or the Wilcoxon rank-sum test for continuous variables.

Univariate linear regression analyses were performed to examine the relationship between disability scores (dependent variable) at 2, 13 and 52 weeks follow-up and age group (independent variable). Afterwards a multivariate linear regression model with all potential predictive variables were performed at each of the follow-up times to examine the relationship between disability and each of the potential predictive variables, adjusted for age group. Finally, variables were entered into a backwards stepwise regression modelling with all relevant baseline variables included in the initial model and a significance level of 0.05 for variable retention. This approach iteratively removes variables with p-values above the threshold to identify the most significant predictors while minimizing overfitting. Separate models were fitted for each of the three follow-ups. Results from the models were reported as regression coefficients (β) with 95% confidence intervals (CIs) and p-values.

Assumptions for linear regression were assessed post-regression. Normality of residuals was evaluated using Q-Q plots, histograms, and the Shapiro–Wilk test. Homoskedasticity was tested using the Breusch-Pagan test, and robust standard errors were applied as appropriate.

In ChiCo missing data in the RMDQ were handled through chained multiple imputation of unanswered items to allow for calculation of sum scores for incomplete questionnaires [22]. For the subsequent analyses, patients with no responses on RMDQ at any timepoint were excluded, as were those with information for fewer than 15 of the 23 items.

No imputation was performed for other variables. The regression models were conducted as complete-case analyses, meaning that participants with missing data on any included variable were excluded from that specific model. Robust standard errors were used to account for heteroscedasticity and model variability due to differing sample sizes.

All statistical analyses were performed using Stata, and significance was set at a p-value of < 0.05.

## Results

A total of 2,848 participants were available at baseline. After excluding 71 due to missing RMDQ scores, the final sample consisted of 2,777 participants, of whom 1,066 were categorized in the young group (< 40 years), 1,270 in the middle-aged group (40–59 years), and 441 in the older group (≥ 60 years).

The mean age of the study population was 44.5 years (95% CI 39.4–43.1), and 41.3% were female. Compared to younger and middle-aged patients, a higher proportion of older patients reported non-MSK comorbidities and use of prescription pain medication. Younger patients exhibited higher levels of depression and anxiety compared to older and middle-aged patients, whereas a higher proportion of younger and middle-aged patients reported three or more MSK comorbidities compared to older patients. There were no major differences between the age groups in back and leg pain intensity or disability scores at baseline (Table 1).

**Table 1:** Patient characteristics stratified by age group

	< 40 years	40–59 years	≥ 60 years	Total
n (%)	1066 (38.4)	1270 (45.7)	441 (15.9)	2777 (100.0)
Sex (female), % (95% CI)	40.6 (37.7 to 43.6)	42.9 (40.2 to 45.7)	38.1 (33.7 to 42.7)	41.3 (39.4 to 43.1)
Missing, n	0	0	0	0
Age, mean (95% CI)	30.4 (30.0 to 30.7)	48.9 (48.6 to 49.2)	65.6 (65.2 to 66.1)	44.5 (44.0 to 45.0)
Missing, n	0	0	0	0
Low back pain (0–10), mean (95% CI)	6.6 (6.5 to 6.8)	6.8 (6.7 to 6.9)	6.5 (6.3 to 6.7)	6.7 (6.6 to 6.8)
Missing, n	10	27	15	52
Leg pain (0–10), median (IQI)	2.0 (0.0 to 5.0)	2.0 (0.0 to 5.0)	3.0 (0.0 to 6.0)	2.0 (0.0 to 5.0)
Missing, n	15	28	15	58
RMDQ (0–100), mean (CI)	53.4 (52.0 to 54.9)	56.5 (55.1 to 57.8)	54.3 (52.2 to 56.4)	55.0 (54.1 to 55.9)
Missing, n	30	27	20	60
BMI in groups, % (95% CI)				
Normalweight	48.2 (44.5 to 52.0)	35.0 (32.0 to 38.0)	37.3 (32.6 to 42.3)	39.8 (37.7 to 42.0)
Overweight	32.3 (29.0 to 36.0)	41.5 (38.4 to 44.5)	41.3 (36.4 to 46.3)	38.4 (36.3 to 40.5)
Obese	19.5 (16.7 to 22.6)	23.6 (21.0 to 26.3)	21.4 (17.6 to 25.9)	21.8 (20.1 to 23.7)
Missing, n	379	286	63	728
Living with partner/spouse (Yes), % (95% CI)	72.4 (69.0 to 75.6)	82.8 (80.3 to 85.1)	86.2 (82.3 to 89.3)	80.0 (78.2 to 81.6)
Missing, n	373	280	64	717
Analgesics use for LBP, % (95% CI)				
No	55.9 (52.2 to 59.6)	46.5 (43.4 to 49.7)	42.6 (37.7 to 47.7)	49 (46.8 to 51.1)
Non-prescription analgesics	31.2 (27.8 to 34.8)	34.8 (31.9 to 37.9)	34.8 (30.1 to 39.8)	33.6 (31.6 to 35.7)
Prescription analgesics	12.9 (10.6 to 15.6)	18.6 (16.3 to 21.2)	22.6 (18.7 to 27.2)	17.4 (15.8 to 19.2)
Missing, n	383	288	70	741
Highest education, % (95% CI)				
No/primary/youth	16.4 (13.8 to 19.4)	15.5 (13.4 to 18.0)	17.2 (13.5 to 21.6)	16.1 (14.5 to 17.8)
Vocational/short	39.9 (36.2 to 43.7)	46.6 (43.5 to 49.8)	41.8 (36.7 to 47.2)	43.5 (41.3 to 45.7)
Middle further	28.7 (25.4 to 32.3)	26.6 (23.9 to 29.6)	32.6 (27.8 to 37.8)	28.4 (26.4 to 30.4)
Higher	15.0 (12.5 to 18.0)	11.2 (9.3 to 13.4)	8.3 (5.8 to 11.8)	12.0 (10.6 to 13.5)
Missing, n	407	324	104	835
Anxiety, median (IQI)	5.0 (2.0 to 7.0)	4.0 (1.0 to 6.0)	2.0 (0.0 to 4.0)	4.0 (1.0 to 6.0)
Missing, n	5	14	11	30
Depression, median (IQI)	3.0 (1.0 to 6.0)	2.0 (0.0 to 5.0)	2.0 (0.0 to 5.0)	2.0 (0.0 to 5.0)
Missing, n	10	18	12	40
Kinesiophobia (High risk), % (95% CI)	45.8 (42.8 to 48.8)	40.9 (38.2 to 43.7)	47.8 (43.0 to 52.6)	43.9 (42.0 to 45.8)
Missing, n	16	40	27	83
Number of MSK comorbidities, % (95% CI)				
0	16.1 (13.5 to 19.1)	15.1 (12.9 to 17.5)	20.4 (16.5 to 24.9)	16.4 (14.8 to 18.1)
1	15.9 (13.3 to 18.9)	17.0 (14.8 to 19.5)	20.4 (16.5 to 24.9)	17.3 (15.7 to 19.0)
2	16.8 (14.2 to 19.9)	17.1 (14.9 to 19.6)	19.3 (15.5 to 23.7)	17.4 (15.8 to 19.2)
3 or more	51.1 (47.3 to 54.9)	50.8 (47.6 to 53.9)	39.9 (35.0 to 45.1)	48.9 (46.7 to 51.1)
Missing, n	401	307	83	791
Number of non-MSK comorbidities, % (95% CI)				
0	63.0 (59.2 to 66.7)	45.7 (42.5 to 49.0)	20.1 (16.3 to 24.6)	46.5 (44.2 to 48.7)
1	28.7 (25.2 to 32.4)	32.3 (29.4 to 35.4)	34.7 (30.0 to 39.8)	31.6 (29.5 to 33.7)
2	6.4 (4.8 to 8.7)	14.5 (12.4 to 16.9)	26.4 (22.2 to 31.2)	14.1 (12.7 to 15.8)
3 or more	1.9 (1.1 to 3.4)	7.5 (5.9 to 9.3)	18.7 (15.0 to 23.1)	7.8 (6.7 to 9.1)
Missing, n	445	345	78	868
General health, % (95% CI)				
Excellent	16.0 (13.1 to 19.4)	14.9 (12.5 to 17.7)	15.3 (11.0 to 20.9)	15.3 (13.6 to 17.3)
Very fine	42.1 (37.9 to 46.4)	40.9 (37.3 to 44.5)	44.5 (37.9 to 51.3)	41.8 (39.3 to 44.4)
Fine	27.9 (24.2 to 31.9)	26.0 (22.9 to 39.3)	22.5 (17.3 to 28.6)	26.2 (24.0 to 28.5)
Fair/Bad	14.0 (11.3 to 17.3)	18.2 (15.6 to 21.2)	17.7 (13.1 to 23.5)	16.7 (14.8 to 18.7)
Missing, n	553	551	232	1336

CI: confidence interval, BMI: body mass index, IQI: interquartile interval, LBP: low back pain, MSK: musculoskeletal, RMDQ: Roland Morris Disability Questionnaire

**Fig. 1:**
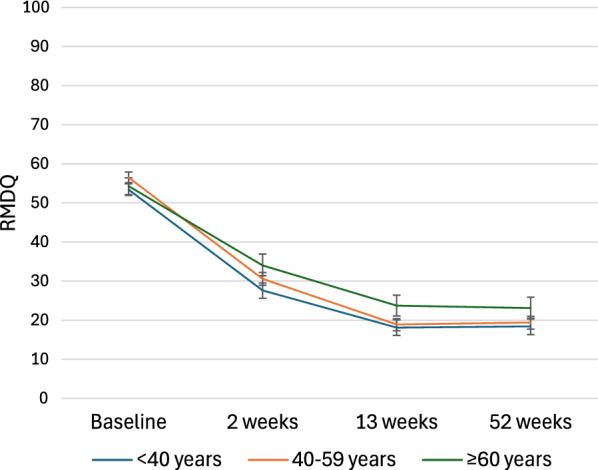
Mean disability scores for the three age groups at baseline and all follow-ups. RMDQ: Roland Morris Disability Questionnaire

Disability scores decreased over time regardless of age group, although older patients had slightly higher scores at all three follow-ups (Fig. 1). Older patients showed slightly less change in disability scores from baseline to all three follow-up points compared with younger and middle-aged patients. (Table 2). No differences were observed between younger- and middle-aged patients (Table 2).

**Table 2:** Mean change and difference in mean change in disability scores

	Baseline to 2-weeks		Baseline to 13-weeks		Baseline to 52-weeks	
	Mean change	Difference	Mean change	Difference	Mean change	Difference
Age						
< 40	− 25.9 (− 23.9 to − 28.0)	–	− 35.7 (− 33.1 to − 38.3)	–	− 35.2 (− 32.5 to − 37.9)	–
40–59	− 26.0 (− 24.4 to − 27.7)	− 0.1 (− 2.8 to 2.5)	− 36.9 (− 35.0 to − 38.8)	− 1.2 (− 4.3 to 1.9)	− 36.5 (− 34.4 to − 38.5)	− 1.3 (− 4.6 to 2.1)
> 60	− 20.3 (− 17.7 to − 22.9)	5.6 (2.2 to 9.0)	− 30.6 (− 27.7 to − 33.5)	5.1 (1.1 to 9.0)	− 30.8 (− 27.6 to − 34.1)	4.4 (0.1 to 8.6)

Numbers presented are means and difference in mean changes with 95% confidence intervals

Of the 13 included variables, 7 remained in the backwards stepwise regression model at 2- and 13 weeks, and 6 at 52 weeks.

Across all follow-ups, higher baseline disability, depression, having three or more MSK comorbidities, and decreasing self-perceived general health were consistently associated with higher disability scores. The association between MSK comorbidities and disability was strongest at 52 weeks, increasing with the number of comorbidities.

Other variables showed less consistent associations. Older patients had higher disability scores at 13 and 52 weeks but not at 2 weeks. Non-MSK comorbidities were associated with higher disability scores at 2 and 13 weeks but not at 52 weeks, with the strongest association seen in those with three or more non-MSK comorbidities. Education level and use of analgesics also showed varying associations at different time points, but effect sizes were modest (Table 3).

**Table 3:** Association between patient characteristics and disability outcomes at baseline and follow-ups

	2 weeks x						52 weeks						52 weeks					
	Full adjusted model			Backwards stepwise model			Full adjusted model			Backwards stepwise model			Full adjusted model			Backwards stepwise model		
	β	95% CI	*p*-value	β	95% CI	*p*-value	β	95% CI	*p*-value	β	95% CI	*p*-value	β	95% CI	*p*-value	β	95% CI	*p*-value
Baseline RMDQ score	0.36	0.30 to 0.42	< 0.01	0.38	0.32 to 0.44	< 0.01	0.17	0.11 to 0.23	< 0.01	–	–	–	0.18	0.12 to 0.25	< 0.01	–	–	–
Age																		
< 40 (ref)	–	–	–	–	–	–	–	–	–	–	–	–	–	–	–	–	–	–
40–59	2.00	− 0.99 to 5.00	0.19	–	–	–	− 0.64	− 3.72 to 2.45	0.69				0.59	− 3.10 to 3.22	0.97	–	–	–
≥ 60	5.08	0.49 to 9.65	0.03	–	–	–	6.64	2.00 to 11.27	< 0.01	7.8	3.7 to 11.81	< 0.01	4.29	− 0.43 to 9.00	0.08	4.81	0.91 to 8.70	0.02
Sex																		
Male (ref)	–	–	–	–	–	–	–	–	–	–	–	–	–	–	–	–	–	–
Female	1.11	− 1.79 to 4.00	0.45	–	–	–	− 1.56	− 4.46 to 1.34	0.29	–	–	–	− 1.79	− 4.74 to 1.17	0.24	–	–	–
BMI																		
Normal weight (ref)	–	–	–	–	–	–	–	–	–	–	–	–	–	–	–	–	–	–
Overweight	2.34	− 0.72 to 5.39	0.14	–	–	–	− 1.40	− 4.42 to 1.61	0.36	–	–	–	2.71	− 0.47 to 5.90	0.10	–	–	–
Obese	2.86	− 0.83 to 6.55	0.13	–	–	–	− 1.28	− 5.26 to 2.69	0.53	–	–	–	0.96	− 2.70 to 4.63	0.61	–	–	–
Living with partner/spouse																		
No (ref)	–	–	–	–	–	–	–	–	–	–	–	–	–	–	–	–	–	–
Yes	1.14	− 2.28 to 4.57	0.51	–	–	–	3.11	− 0.19 to 6.41	0.07	–	–	–	− 0.21	− 3.91 to 3.50	0.91			
Anlgesics use for LBP																		
No (ref)	–	–	–	–	–	–	–	–	–	–	–	–	–	–	–	–	–	–
Non− prescription	1.91	− 1.29 to 5.12	0.24	–	–	–	− 2.03	− 5.23 to 1.17	0.21	− 3.30	− 6.28 to − 0.32	0.04	− 1.71	− 5.00 to 1.59	0.31	–	–	–
Prescription	5.09	0.77 to 9.42	0.02	4.57	0.54 to 8.60	0.03	3.23	− 1.22 to 7.68	0.15	–	–	–	− 1.10	− 5.31 to 3.11	0.61	–	–	–
Highest education																		
No/primary/youth (ref)	–	–	–	–	–	–	–	–	–	–	–	–	–	–	–	–	–	–
Vocational/short	− 3.31	− 7.60 to 0.38	0.08	− 2.95	− 5.63 to − 0.26	0.03	− 4.84	− 9.69 to 0.01	0.05	–	–	–	− 4.94	− 9.93 to 0.06	0.05	–	–	–
Middle further	− 1.51	− 5.74 to 2.73	0.49	–	–	–	− 3.20	− 8.19 to 1.79	0.21	–	–	–	− 3.21	− 8.39 to 1.98	0.23	–	–	–
Higher	1.87	− 3.55 to 7.29	0.50	–	–	–	− 2.21	− 7.99 to 3.58	0.45	–	–	–	3.27	− 9.27 to 2.73	0.29	–	–	–
Anxiety	− 0.25	− 0.81 to 0.31	0.39	–	–	–	− 0.10	− 0.65 to 0.44	0.71	–	–	–	0.28	− 0.27 to 0.82	0.32	–	–	–
Depression	1.06	0.48 to 1.63	< 0.01	0.90	0.39 to 1.41	< 0.01	0.86	0.32 to 1.40	< 0.01	0.87	0.35 to 1.38	< 0.01	0.81	0.21 to 1.4	0.01	0.99	0.45 to 1.52	< 0.01
Kinesiophobia																		
Low risk (ref)	–	–	–	–	–	–	–	–	–	–	–	–	–	–	–	–	–	–
High risk	2.57	− 0.28 to 5.42	0.08	–	–	–	0.76	− 2.11 to 3.64	0.60	–	–	–	1.70	− 1.20 to 4.61	0.25	–	–	–
Number of MSK comorbidities																		
0 (ref)	–	–	–	–	–	–	–	–	–	–	–	–	–	–	–	–	–	–
1	− 2.16	− 6.71 to 2.40	0.35	–	–	–	2.76	− 1.69 to 7.22	0.22	–	–	–	4.87	1.01 to 8.73	0.01	5.27	1.53 to 9.00	< 0.01
2	− 1.40	− 6.15 to 3.34	0.56	–	–	–	1.89	− 2.37 to 6.15	0.38	–	–	–	8.49	4.10 to 12.89	< 0.01	8.84	4.46 to 13.21	< 0.01
3 or more	3.00	− 1.44 to 7.43	0.19	3.98	1.13 to 6.84	< 0.01	6.75	2.54 to 10.96	< 0.01	4.86	1.99 to 7.74	< 0.01	11.35	7.50 to 15.20	< 0.01	11.54	8.05 to 15.03	< 0.01
Number of non− MSK comorbidities																		
0 (ref)	–	–	–	–	–	–	–	–	–	–	–	–	–	–	–	–	–	–
1	− 0.87	− 4.01 to 2.28	0.59	–	–	–	− 0.68	− 3.67 to 2.32	0.66	–	–	–	− 0.45	− 3.64 to 2.75	0.78	–	–	–
2	4.26	− 0.18 to 8.70	0.06	5.93	1.87 to 9.99	< 0.01	7.39	2.55 to 12.25	< 0.01	7.67	3.11 to 12.24	< 0.01	1.79	− 2.99 to 6.55	0.46	–	–	–
3 or more	7.09	0.75 to 13.44	0.03	8.74	2.81 to 14.67	< 0.01	7.54	0.31 to 14.77	0.04	7.07	0.10 to 14.04	0.04	6.47	− 0.15 to 13.10	0.06	–	–	–
General health																		
Excellent (ref)	–	–	–	–	–	–	–	–	–	–	–	–	–	–	–	–	–	–
Very fine	6.41	2.66 to 10.16	< 0.01	6.30	2.57 to 9.99	< 0.01	1.93	− 1.90–5.76	0.32	–	–	–	1.02	− 2.91 to 4.95	0.61	–	–	–
Fine	9.30	5.02 to 13.60	< 0.01	9.22	5.05 to 13.39	< 0.01	7.43	2.98–11.88	< 0.01	6.07	2.88 to 9.26	< 0.01	6.94	2.19 to 11.69	< 0.01	6.89	3.49 to 10.30	< 0.01
Fair/Bad	10.78	5.59 to 15.95	< 0.01	10.75	5.55 to 15.95	< 0.01	13.47	8.79 to 18.15	< 0.01	13.43	8.77 to 18.10	< 0.01	12.01	6.19 to 17.83	< 0.01	12.92	8.37 to 17.48	< 0.01

Multiple linear regression models showing the association between patient symptoms and characteristics and RMDQ. BMI: body mass index, CI: confidence interval, LBP: low back pain, MSK: musculoskeletal, Ref: reference category, RMDQ: Roland Morris Disability Questionnaire

## Discussion

This study found that older patients in chiropractic care had a higher prevalence of non-MSK comorbidities and more frequent use of prescription pain medication compared to younger and middle-aged patients. In contrast, younger and middle-aged patients reported a higher prevalence of MSK comorbidities and younger patients also exhibited higher levels of depression and anxiety compared to older patients. While older patients reported slightly higher disability scores and less improvement over time, age alone was not the strongest factor associated with disability. Instead, baseline disability, depression, self-perceived general health, and MSK comorbidities were more consistently associated with higher disability scores across all follow-up times. These findings suggest that older age alone may not be the primary driver of prolonged disability, but that other demographic and health-related factors might play a bigger role in disability outcomes.

Our findings align with Manogharan et al.[33], who examined the impact of age on pain and disability outcomes in patients from a secondary care spine clinic with chronic LBP over one year. The study showed that older patients had slightly higher disability scores at all time points but showed comparable improvement over time to younger patients [33]. While the oldest patients exhibited slightly less improvement than the youngest group, the differences were small, and there was no clear evidence that age itself significantly influenced changes in disability.

Also, a Norwegian primary care study (BACE-N) [34], which included patients aged 55 years and older who consulted GPs, physiotherapists, and chiropractors, found that psychological symptoms and comorbidity profiles were associated with disability, and that these patterns were largely consistent across professions. Although our study focused only on chiropractic patients, the observed associations align closely with those reported in BACE-N [34]. Our study showed that a higher number of comorbidities, especially MSK comorbidities, was associated with higher disability scores at all three follow-up points. This is consistent with a study by Fu et al. [35] on GP patients with LBP aged 55 and older that found that a greater number of MSK comorbidities were associated with higher pain- and disability scores at 3- and 12 months follow up. We found that a significantly higher proportion of younger patients reported three or more MSK comorbidities at baseline compared with older patients, which contrary to other literature that reported that older patients were more likely to report higher numbers of both MSK and non-MSK comorbidities, compared to younger patients [7, 8, 36].

***The well-being paradox****:* Older patients might underreport symptoms, seeing pain as a normative part of aging, while younger patients may perceive pain as more disruptive, leading to greater psychological strain and higher comorbidity counts. This is described as the "well-being paradox" which states that despite older adults being in higher risk of disability, their well-being and psychological health is not worse than in younger adults [37, 38]. In fact, our study found that younger patients reported significantly higher anxiety and depression scores than middle-aged and older patients. This is also described in a study by Wettstein et al. [39], where they found that older patients reported lower anxiety and depression, and better self-perceived physical health than younger patients despite higher disability scores. However, this contrasts with a study by Scheele et al., in which they found that older patients experienced more symptoms of depression than their younger group [8]. These discrepancies might be explained by the differences in study populations, with Wettstein et al. examining patients from a specialised spine centre and Scheele et al. examining a population of GP patients.

Our study population consisted of chiropractic patients, and previous research has questioned whether they are representative of the wider LBP population due to notable differences compared to, for example, GP patients. A study by Hestbæk et al. [40] found that GP patients typically present with more severe symptoms, including higher pain intensity, longer pain duration, greater disability, and more psychosocial factors such as depression and fear-avoidance compared to chiropractic patients. These differences raise questions about whether chiropractic patients are generally healthier than the broader LBP population.

Given that MSK comorbidities and depression were among the most consistent factors associated with higher disability scores at all three follow-up points in our study, this may help to explain why age alone did not seem to have the most significant impact on disability. Our findings might therefore suggest potential benefits of considering a biopsychosocial approach in the management of LBP.

While older patients in our study showed slightly less improvement in disability scores compared to younger groups, the absolute differences between age groups were relatively small. Prior research has suggested that a minimal clinically important difference (MCID) for the original 0–23 RMDQ scale is approximately 38% change, equivalent to roughly 17–22 points on a 0–100 scale [41]. The observed differences in our study were therefore well below this threshold at all follow-up time points, suggesting that the between-group differences in disability improvement are not of meaningful clinical importance. This finding reinforces the interpretation that age, although associated with disability outcomes, may play a limited role in determining whether patients experience a clinically relevant improvement.

## Limitations

The data from the ChiCo cohort were collected from 10 chiropractic clinics. There is a theoretical risk that these clinics are not representative of Danish chiropractic clinics. However, Danish chiropractic clinics are relatively homogeneous and there is no indication that these were not representative of Danish chiropractic clinics, as baseline demographics were reported previously to be similar to those found in a Danish national cohort of chiropractic patients with LBP [22].

Danish chiropractic patients with LBP are not representative of the broader population of Danish primary care patients with LBP. Research indicates that they tend to be younger, predominantly male, better educated, and in better overall health with fewer comorbidities than those who seek treatment for LBP from Danish general practitioners [40].

The ChiCo cohort used brief screening questions rather than full validated scales to assess depression, anxiety, and kinesiophobia. While this approach facilitated data collection in a pragmatic clinical setting, it may have increased the risk of misclassification and residual confounding in the multivariable models. However, similar brief items for depression and anxiety have previously been used [30].

Our study included a greater number of younger patients than older patients. Despite our efforts to combine or omit subgroups with very few patients, some potential prognostic variables still had relatively few observations in certain categories compared to others. This can cause issues with overfitting due to high variance in small groups, which can bias the results and potentially lead to less reliable estimates.

A substantial proportion of participants had missing data on one or more of the variables included in the regression models, resulting in reduced sample sizes in the adjusted analyses, with the* “general health*” variable having the largest proportion of missing data (1,336 of 2,777). However, we chose to retain the variable in the final models because of its theoretical and clinical relevance as a holistic indicator of patient health. To assess the impact of missing data, we conducted sensitivity analyses excluding this variable (Supplementary file 1, Table S3). These analyses showed that the overall pattern of associations remained largely consistent, with certain associations becoming slightly stronger. Despite these limitations, our study also has several strengths. It is based on a large cohort of chiropractic patients with LBP, enhancing its external validity within chiropractic settings. Additionally, the study benefits from a long follow-up period on 52 weeks, allowing for an investigation of both short- and long-term outcomes.

## Conclusion

This study found that while older chiropractic patients with LBP had slightly higher disability scores and a higher prevalence of non-MSK comorbidities, age was not the strongest factor associated with disability outcomes. Instead, baseline disability, depression, self-perceived general health, and MSK comorbidities were more consistently linked to higher disability scores across all follow-up time points. These findings suggest that age should not be viewed as an isolated risk factor for prolonged disability, but rather as one part of a broader biopsychosocial framework.

## Supplementary Information

Supplementary Materials

Supplementary Materials

## Data Availability

No datasets were generated or analysed during the current study.

## Abbreviations

BMI: Body mass index
ChiCo: Danish chiropractic low back pain cohort
CI: Confidence interval
CPR: Central person register
GP: General practitioner
IQI: Interquartile interval
LBP: Low back pain
MSK: Musculoskeletal
NRS: Numeric rating scale
OMPQ: Örebro musculoskeletal pain screening questionnaire
RMDQ: Roland morris disability questionnaire
TSK: Tampa scale for kinesiophobia
