# Supplementary material for: Differences in demographics and clinical outcomes in older, middle-aged, and younger adults with low back pain receiving chiropractic care
Source: Chiropr Man Therap. 2025 Jul 31;33:31. doi: 10.1186/s12998-025-00589-w (PMC12315371; doi:10.1186/s12998-025-00589-w)
Supplement: Supplementary file 1 — Supplementary Materials [file 12998_2025_589_MOESM1_ESM.docx]

| Did a doctor ever tell you that you have or have had diabetes? | 1 = Yes |
| --- | --- |
| Did a doctor ever tell you that you have or have had osteoporosis? | 1 = Yes |
| Did a doctor ever tell you that you have or have had thrombus in the hearth, brain or somewhere else? | 1 = Yes |
| Did a doctor ever tell you that you have or have had hypertension? | 1 = Yes |
| Did a doctor ever tell you that you have or have had psoriasis? | 1 = Yes |
| Did a doctor ever tell you that you have or have had rheumatoid arthritis? | 1 = Yes |
| Did a doctor ever tell you that you have or have had osteoarthritis? | 1 = Yes |
| Did a doctor ever tell you that you have or have had fibromyalgia? | 1 = Yes |
| Did a doctor ever tell you that you have or have had metabolic diseases (too high or too low metabolism)? | 1 = Yes |
| Did a doctor ever tell you that you have or have had asthma? | 1 = Yes |
| Did a doctor ever tell you that you have or have had migraine? | 1 = Yes |
| Did a doctor ever tell you that you have or have had chronic inflammatory bowel disease (Crohn's disease)? | 1 = Yes |
| Did a doctor ever tell you that you have or have had cancer? | 1 = Yes |
| Did a doctor ever tell you that you have or have had COPD or chronic bronchitis? | 1 = Yes |
| Did a doctor ever tell you that you have or have had a neurological disease (e.g. sclerosis or Parkinson's disease)? | 1 = Yes |
| None of the diseases mentioned | 1 = Yes |

Table S1 - Questions regarding non-MSK comorbidities

*MSK: Musculoskeletal*

Table S2 - Questions regarding MSK comorbidities

| In addition to LBP, did you within the last 2 weeks experience headache? | 1 = Yes |
| --- | --- |
| In addition to LBP, did you within the last 2 weeks experience pain in the neck? | 1 = Yes |
| In addition to LBP, did you within the last 2 weeks experience chest pain ? | 1 = Yes |
| In addition to LBP, did you within the last 2 weeks experience pain in the stomach? | 1 = Yes |
| In addition to LBP, did you within the last 2 weeks experience shoulder pain? | 1 = Yes |
| In addition to LBP, did you within the last 2 weeks experience pain in the elbows? | 1 = Yes |
| In addition to LBP, did you within the last 2 weeks experience pain in the arms? | 1 = Yes |
| In addition to LBP, did you within the last 2 weeks experience pain in the hands? | 1 = Yes |
| In addition to LBP, did you within the last 2 weeks experience hip pain? | 1 = Yes |
| In addition to LBP, did you within the last 2 weeks experience knee pain? | 1 = Yes |
| In addition to LBP, did you within the last 2 weeks experience leg pain? | 1 = Yes |
| In addition to LBP, did you within the last 2 weeks experience pain in the feet? | 1 = Yes |
| In addition to LBP, did you within the last 2 weeks experience pain anywhere else? | 1 = Yes |

*MSK: Musculoskeletal*

|  | 2 weeks | | |  | 13 weeks | | |  | 52 weeks | | |
| --- | --- | --- | --- | --- | --- | --- | --- | --- | --- | --- | --- |
|  | **β** | **95% CI** | **p-value** |  | **β** | **95% CI** | **p-value** |  | **β** | **95% CI** | **p-value** |
| Baseline RMDQ score | 0.39 | 0.34 to 0.44 | <0.01 |  | 0.18 | 0.13 to 0.23 | <0.01 |  | 0.19 | 0.14 to 0.24 | <0.01 |
| Age |  |  |  |  |  |  |  |  |  |  |  |
| <40 (ref) | - | - | - |  | - | - | - |  | - | - | - |
| 40-59 | - | - | - |  |  |  |  |  | - | - | - |
| ≥60 | 3.42 | 0.19 to 6.66 | 0.04 |  | 5.25 | 2.03 to 8.47 | <0.01 |  | 5.82 | 2.54 to 9.11 | <0.01 |
| Sex |  |  |  |  |  |  |  |  |  |  |  |
| Male (ref) | - | - | - |  | - | - | - |  | - | - | - |
| Female | - | - | - |  | - | - | - |  | - | - | - |
| BMI |  |  |  |  |  |  |  |  |  |  |  |
| Normal weight (ref) | - | - | - |  | - | - | - |  | - | - | - |
| Overweight | - | - | - |  | - | - | - |  | 3.28 | 0.60 to 5.97 | 0.02 |
| Obese | - | - | - |  | - | - | - |  | 3.93 | 0.66 to 7.20 | 0.02 |
| Living with partner/spouse |  |  |  |  |  |  |  |  |  |  |  |
| No (ref) | - | - | - |  | - | - | - |  | - | - | - |
| Yes | - | - | - |  | - | - | - |  |  |  |  |
| Anlgesics use for LBP |  |  |  |  |  |  |  |  |  |  |  |
| No (ref) | - | - | - |  | - | - | - |  | - | - | - |
| Non-prescription | - | - | - |  | - | - | - |  | - | - | - |
| Prescription | 5.32 | 1.91 to 8.74 | <0.01 |  | 4.95 | 1.41 to 8.49 | <0.01 |  | - | - | - |
| Highest education |  |  |  |  |  |  |  |  |  |  |  |
| No/primary/youth (ref) | - | - | - |  | - | - | - |  | - | - | - |
| Vocational/short | 2.70 | -5.01 to -0.39 | 0.02 |  | - | - | - |  | - | - | - |
| Middle further | - | - | - |  | - | - | - |  | - | - | - |
| Higher | - | - | - |  | - | - | - |  | - | - | - |
| Anxiety | - | - | - |  | - | - | - |  | - | - | - |
| Depression | 1.20 | 0.76 to 1.65 | <0.01 |  | 1.23 | 0.79 to 1.68 | <0.01 |  | 1.29 | 0.82 to 1.78 | <0.01 |
| Kinesiophobia |  |  |  |  |  |  |  |  |  |  |  |
| Low risk (ref) | - | - | - |  | - | - | - |  | - | - | - |
| High risk | 2.59 | 0.16 to 5.02 | 0.04 |  | - | - | - |  | - | - | - |
| Number of MSK  comorbidities |  |  |  |  |  |  |  |  |  |  |  |
| 0 (ref) | - | - | - |  | - | - | - |  | - | - | - |
| 1 | - | - | - |  | - | - | - |  | 5.19 | 1.86 to 8.5 | <0.01 |
| 2 | - | - | - |  | - | - | - |  | 9.55 | 5.80 to 13.29 | <0.01 |
| 3 or more | 5.47 | 3.11 to 7.84 | <0.01 |  | 7.96 | 5.54 to 10.38 | <0.01 |  | 13.38 | 10.42 to 16.33 | <0.01 |
| Number of non-MSK  comorbidities |  |  |  |  |  |  |  |  |  |  |  |
| 0 (ref) | - | - | - |  | - | - | - |  | - | - | - |
| 1 | - | - | - |  | - | - | - |  | - | - | - |
| 2 | 6.98 | 3.42 to 10.53 | <0.01 |  | 8.61 | 4.65 to 12.56 | <0.01 |  | - | - | - |
| 3 or more | 8.91 | 3.92 to 13.90 | <0.01 |  | 11.6 | 5.54 to 17.64 | <0.01 |  | 9.95 | 4.17 to 15.74 | <0.01 |

Table S3 – Sensitivity analysis: *Association between patient characteristics and disability outcomes at baseline and follow-ups* ***without general health***

*BMI: body mass index, CI: confidence interval, LBP: low back pain, MSK: musculoskeletal, Ref: reference category, RMDQ: Roland Morris Disability Questionnaire.*
